# Stress analysis and applicability analysis of the elliptical head

**DOI:** 10.1038/s41598-021-02397-7

**Published:** 2021-11-25

**Authors:** Zhanhui Wang, Zhifang Zhang, Jinzhong Chen, Jinjun Bai

**Affiliations:** 1 School of Chemistry and Chemical Engineering, Yulin University, Yulin, 719000 Shanxi China; 2 Key Laboratory of Low Metamorphic Coal Clean Utilization, Yulin, 719000 Shanxi China

**Keywords:** Energy science and technology, Engineering, Materials science

## Abstract

As the main pressure components of pressure vessels, the mechanical performance of cylinders and heads affects the normal operation of pressure vessels. At present, no unified theoretical formula exists for the connection region between an elliptical head and the cylinder. Therefore, the authors consider the standard elliptical head as the research object. First, the theoretical stress calculation formula is deduced according to the deformation continuity equation. Second, the stress is experimentally measured using an internal-pressure thin-walled-vessel stress measurement device, and the theoretical and experimental stress values in the discontinuous region between the elliptical head and cylinder are analysed and compared to verify the accuracy and applicability of the theoretical stress calculation formula. The results show that the theoretical stress calculation formula in the discontinuous region between the elliptical head and cylinder is valid. By comparing and analysing the theoretical and experimental stress values, the accuracy and applicability of the theoretical stress calculation formula in the discontinuous region are verified. The findings can provide guidance for the stress measurement of internal-pressure vessels.

## Introduction

With the continuous progress of science and technology and the economy, the role of chemical products in people's daily life is becoming increasingly prominent^1^. Processes in the chemical industry are generally characterized by high temperatures, pressures, and corrosivity; therefore, pressure vessels are widely used^2^. According to the magnitude of the pressure, pressure vessels can be divided into internal and external pressure vessels^3,4^. In the chemical industry, internal-pressure vessels are commonly used. As the main pressure bearing components, the mechanical performance of cylinders and heads affects the normal use of pressure vessels^5^. In particular, a pressure vessel bears the combined action of the internal pressure, edge force and edge moment, which causes the stress to increase in the connection region between the cylinder and head. This phenomenon is known as the discontinuous effect: the total stress near the connection region between the cylinder and head increases, rendering it prone to strength failure^6–10^. In this context, analysing the stress near the connection between the cylinder and head is of economic and social significance^11,12^.

Under internal pressure, the cylinder and head individually bear only the membrane stress^13,14^. Under membrane stress, the corresponding membrane deformation is generated, which is examined in terms of the parallel circular radial displacement and meridian angle^15^. This research method is simple and reliable and pertains to the non-moment theory. The non-moment theory is applied considering that the mid plane curvature of the shell has no sudden change, and the boundary is not affected by the transverse shear, bending moment or torque. However, the curvature radii of the cylinder and head are different, and if the cylinder and head are regarded as free bodies, the parallel circular radial displacement and meridian angle differ at the connection boundary under the action of internal pressure. Notably, the cylinder and head are welded together; in other words, at the connecting boundary, the parallel circular radial displacement and meridian angle must be identical, leading to a restraint caused by the transverse shear and bending moment at the connection boundary^16–19^. This scenario does not satisfy the application conditions of the non-moment theory, and thus, the moment theory must be adopted.

The elliptical head is composed of a half ellipsoid and straight edge section^20^. Scholars have performed considerable research on the internal stress distribution of the elliptical head, specifically, to compare the membrane stress with finite element analysis or experimental results; however, this comparison is not entirely accurate^21–23^. First, the elliptical head is only studied as a free body without considering the influence of the edge force and edge moment on the stress distribution. Second, the influence of only the internal pressure is considered, and that of the bending internal force, such as the transverse shear and bending moment, is ignored. Therefore, the moment theory should be used to perform the stress analysis because it considers not only internal membrane forces such as the normal force but also internal bending forces such as the transverse shear and bending moment. The influence of the internal bending force can be ignored far from the boundary of the elliptical head and cylinder. In the area near the boundary, the internal bending force must be considered, that is, moment theory must be used. In most of the existing studies, the moment theory has not been used to determine the stress. In addition, no unified theoretical calculation formula exists for the connection region between the elliptical head and cylinder^24–27^.

Therefore, in this study, the standard elliptical head is considered the research object. First, the theoretical stress calculation formula in the discontinuous region between the elliptical head and cylinder is deduced according to the deformation continuity equation^28^. Second, the experimental stress is measured using an internal-pressure thin-walled vessel stress measurement experimental device, and the theoretical and experimental stress values in the discontinuous region are compared and analysed to verify the accuracy and applicability of the theoretical stress calculation formula in the discontinuous region between the elliptical head and cylinder^19,29^. The findings are expected to have guiding significance for the stress measurement of internal-pressure vessels.

## Theoretical stress calculation formula in the discontinuous region between the elliptical head and cylinder

First, the edge force *Q*_0_ and edge moment *M*_0_ are calculated according to the deformation continuity equation^30^, and subsequently, the internal force and internal moment are determined according to the edge force *Q*_0_ and edge moment *M*_0_. Finally, the theoretical stress calculation formula in the discontinuous region between the elliptical head and cylinder can be obtained by substituting the internal pressure, internal force and internal moment into the calculation formula of the membrane stress and bending stress^31^. The specific analysis process is described in the following text.

### Solution of the edge force ***Q***_0_ and edge moment ***M***_0_ in the discontinuous region of the elliptical head

**Figure 1:**
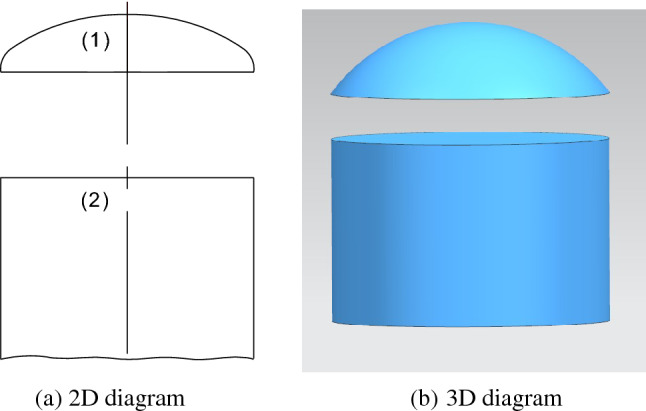
Structural diagram of the elliptical head.

From the perspective of stress and manufacturing, an elliptical head represents the ideal structural form and is widely used in the petroleum, chemical, medicine, machinery, construction, nuclear power and other industries. The connection structure between the elliptical head and cylinder is shown in Fig. 1.

**Figure 2:**
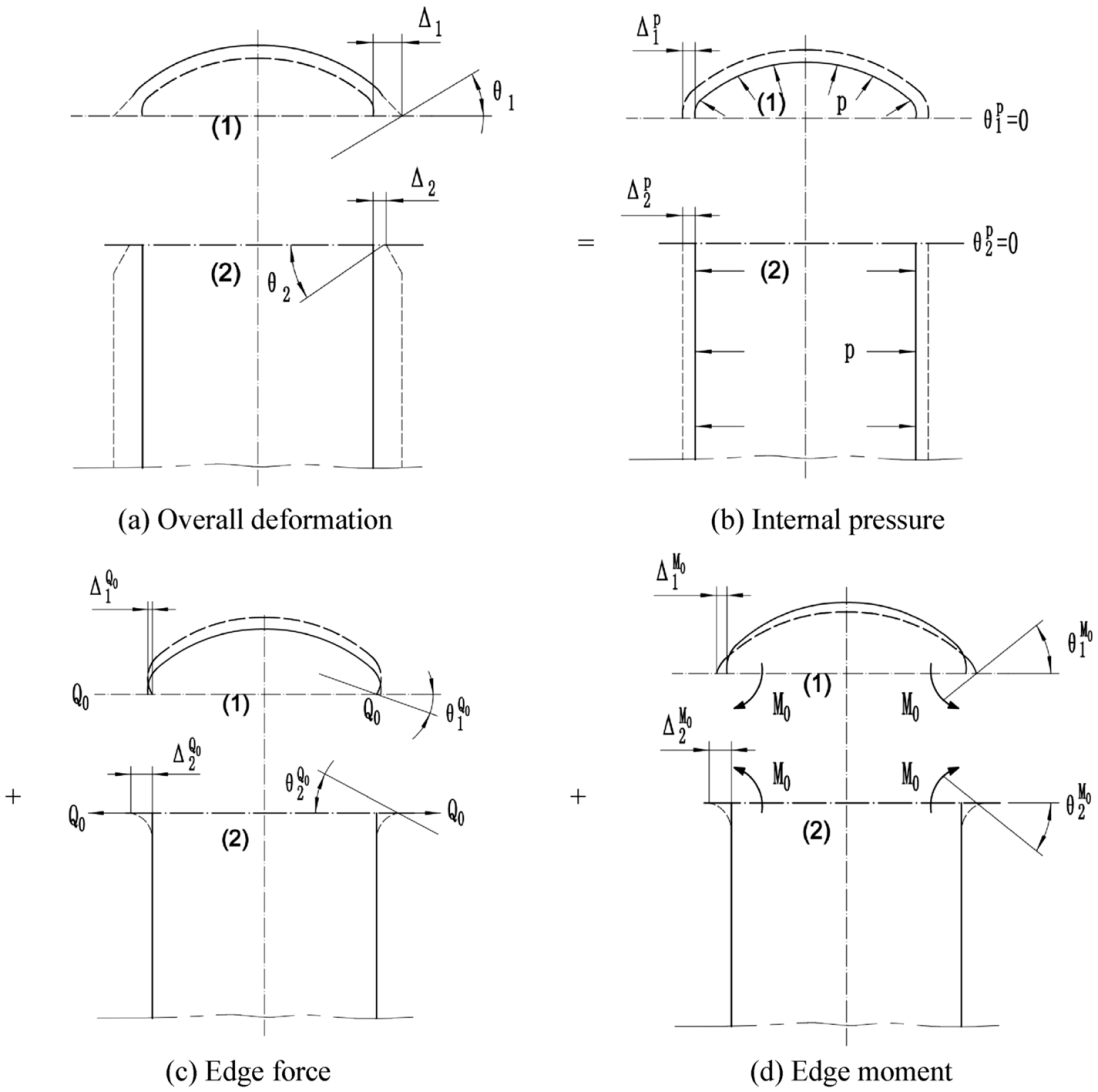
Deformation diagram of the connection boundary.

The connection boundary deformation diagram is shown in Fig. 2, in which (a) shows the overall deformation diagram, (b) shows the membrane deformation caused by the internal pressure *P*, (c) shows the bending deformation caused by the edge force *Q*_0_, and (d) shows the bending deformation caused by the edge moment *M*_0_. According to the deformation continuity condition, after the superposition of the membrane deformation (b) and bending deformation (c) and (d), the total deformation values (a) of the elliptical head and cylinder at the connection boundary are equal.

Under the action of the edge force and edge moment, the rigid body motions of the elliptical head and cylinder are constrained, and they are viewed as rigid bodies. The deformation continuity equation of the elliptical head and the cylinder can be expressed as

1 $$ \left\{ {\begin{array}{l} {\Delta_{1}^{{\text{P}}} + \Delta_{1}^{{{\text{Q}}_{0} }} + \Delta_{1}^{{{\text{M}}_{0} }} = \Delta_{2}^{{\text{P}}} + \Delta_{2}^{{{\text{Q}}_{0} }} + \Delta_{2}^{{{\text{M}}_{0} }} ,} \\ {\theta_{1}^{{\text{P}}} + \theta_{1}^{{{\text{Q}}_{0} }} + \theta_{1}^{{{\text{M}}_{0} }} = \theta_{2}^{{\text{P}}} + \theta_{2}^{{{\text{Q}}_{0} }} + \theta_{2}^{{{\text{M}}_{0} }} .} \\ \end{array} } \right. $$

where subscripts 1 and 2 represent the elliptical head and cylinder, respectively. Superscripts *P*, *Q*_0_ and *M*_0_ represent the internal pressure, edge force and edge moment, respectively. $\Delta_{1}^{{\text{p}}}$, $\theta_{1}^{{\text{p}}}$, $\Delta_{1}^{{{\text{Q}}_{0} }}$, $\theta_{1}^{{{\text{Q}}_{0} }}$, $\Delta_{1}^{{{\text{M}}_{0} }}$, and $\theta_{1}^{{{\text{M}}_{0} }}$ represent the parallel circular radial displacement and meridian angle produced by the elliptical head under the action of the internal pressure *P*, edge force *Q*_0_ and edge moment *M*_0_. $\Delta_{2}^{{\text{p}}}$, $\theta_{2}^{{\text{p}}}$, $\Delta_{2}^{{{\text{Q}}_{0} }}$, $\theta_{2}^{{{\text{Q}}_{0} }}$, $\Delta_{2}^{{{\text{M}}_{0} }}$, and $\theta_{2}^{{{\text{M}}_{0} }}$ denote the parallel circular radial displacement and meridian angle produced by the cylinder under the action of the internal pressure *P*, edge force *Q*_0_ and edge moment *M*_0_.

According to the literature^13^, the membrane deformation equations of the elliptical head can be expressed as

2 $$ \left\{ {\begin{array}{*{20}l} {\Delta_{1}^{{\text{p}}} = P\text{a}^{2} (2 - a^{2} /b^{2} - \mu )/(2Et_{1} ),} \hfill \\ {\theta_{1}^{{\text{p}}} = 0.} \hfill \\ \end{array} } \right. $$

where *t*_1_ is the thickness of the elliptical head, *a* is the length of the semi-major axis, and *b* is the length of the semi-minor axis. *P* is the internal pressure, *μ* is Poisson's ratio, and *E* is the elastic modulus.

The bending deformation equations of the elliptical head can be expressed as

3 $$ \left\{ {\begin{array}{*{20}l} {\Delta_{1}^{{{\text{Q}}_{0} }} = 2\sqrt[4]{{3(1 - \mu^{2} )}}\sqrt {R/t{}_{1}} RQ_{0} /(Et_{1} ),} \hfill \\ {\theta_{1}^{{{\text{Q}}_{0} }} = - 2\sqrt {3(1 - \mu^{2} )} RQ_{0} /(Et_{1}^{2} ),} \hfill \\ {\Delta_{1}^{{{\text{M}}_{0} }} = 2\sqrt {3(1 - \mu^{2} )} RM_{0} /(Et_{1}^{2} ),} \hfill \\ {\theta_{1}^{{{\text{M}}_{0} }} = - 4[3(1 - \mu^{2} )]^{3/4} \sqrt {R/t_{1} } M_{0} /(Et_{1}^{2} ).} \hfill \\ \end{array} } \right. $$

where *R* is the second radius of curvature at the boundary.

The membrane deformation equations of the cylinder can be expressed as

4 $$ \left\{ {\begin{array}{*{20}l} {\Delta_{2}^{{\text{P}}} = PR^{2} (2 - \mu )/(2Et_{2} ),} \hfill \\ {\theta_{2}^{{\text{P}}} = 0.} \hfill \\ \end{array} } \right. $$

where *t*_2_ is the thickness of the cylinder.

The bending deformation equations of the cylinder can be expressed as

5 $$ \left\{ {\begin{array}{*{20}l} {\Delta_{2}^{{{\text{Q}}_{0} }} = - 2^{4} \sqrt {3(1 - \mu^{2} )} \sqrt {R/t_{2} } RQ_{0} /(Et_{2} ),} \hfill \\ {\theta_{2}^{{{\text{Q}}_{0} }} = - 2\sqrt {3(1 - \mu^{2} )} RQ_{0} /(Et_{2}^{2} ),} \hfill \\ {\Delta_{2}^{{{\text{M}}_{0} }} = 2\sqrt {3(1 - \mu^{2} )} RM_{0} /(Et_{2}^{2} ),} \hfill \\ {\theta_{2}^{{{\text{M}}_{0} }} = 4[3(1 - \mu^{2} )]^{3/4} \sqrt {R/t_{2} } M_{0} /(Et_{2}^{2} ).} \hfill \\ \end{array} } \right. $$

When *t*_1_ = *t*_2_, substituting Eqs. (2), (3), (4) and (5) into Eq. (1) yields the edge force and edge moment as follows:

6 $$ \left\{ {\begin{array}{*{20}l} {Q_{0} = \frac{{P\sqrt {Rt} }}{{8^{4} \sqrt {3(1 - \mu^{2} )} }}\left( \frac{a}{b} \right)^{2} ,} \hfill \\ {M_{0} = 0.} \hfill \\ \end{array} } \right. $$

### Solution of the theoretical stress in the discontinuous region of the elliptical head

**Figure 3:**
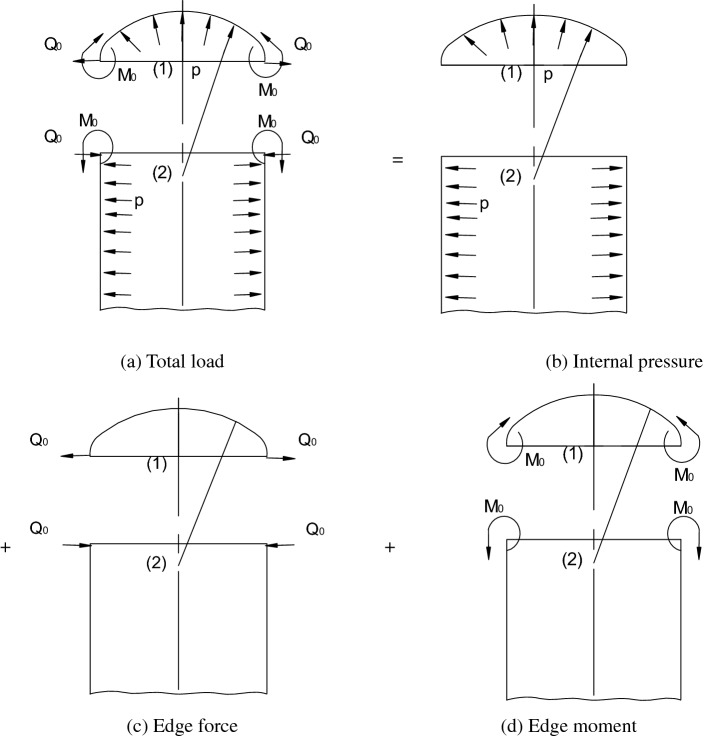
Deformation diagram of the connection boundary.

The connection boundary load diagram is shown in Fig. 3, in which (a) is the overall load diagram, (b) is the diagram of the load caused by the internal pressure *P*, (c) is the diagram of the load caused by the edge force *Q*_0_, and (d) is the diagram of the load caused by the edge moment *M*_0_. The internal pressure *P* produces the internal membrane force, which generates the membrane stress, and the edge force *Q*_0_ and edge moment *M*_0_ produce the internal bending force, which generates the bending stress. The total stress can be achieved by superposition of the membrane stress and bending stress.

According to the literature^30–32^, the total stress in the discontinuous region can be divided into the membrane stress and bending stress, and the theoretical stress equation of the elliptical head can be expressed as

7 $$ \left\{ {\begin{array}{*{20}c} {\sigma_{\upomega } = \sigma_{\upomega }^{{\text{P}}} + N_{\upomega }^{{({\text{Q}}_{0} + {\text{M}}_{0} )}} /t \pm 6M_{\upomega }^{{({\text{Q}}_{0} + {\text{M}}_{0} )}} /t^{2} ,} \\ {\sigma_{\uptheta } = \sigma_{\uptheta }^{{\text{P}}} + N_{\uptheta }^{{({\text{Q}}_{0} + {\text{M}}_{0} )}} /t \pm 6M_{\uptheta }^{{({\text{Q}}_{0} + {\text{M}}_{0} )}} /t^{2} .} \\ \end{array} } \right. $$

where subscripts *θ* and *ω* correspond to the tangential direction of the parallel circle and longitude, respectively. *σ*_ω_ and *σ*_θ_ denote the total longitudinal stress and circumferential stress, respectively. $\sigma_{\upomega }^{{\text{P}}}$ and $\sigma_{\uptheta }^{{\text{P}}}$ denote the longitudinal stress and circumferential stress under internal pressure *P*, respectively. $N_{\upomega }^{{{\text{Q}}_{0} }}$, $N_{\uptheta }^{{{\text{Q}}_{0} }}$, $N_{\upomega }^{{{\text{M}}_{0} }}$, and $N_{\uptheta }^{{{\text{M}}_{0} }}$ are the internal forces along the tangential direction of the longitude and parallel circle under the action of the edge force *Q*_0_ and edge moment *M*_0_; $M_{\upomega }^{{{\text{Q}}_{0} }}$, $M_{\uptheta }^{{{\text{Q}}_{0} }}$, $M_{\upomega }^{{{\text{M}}_{0} }}$, and $M_{\uptheta }^{{{\text{M}}_{0} }}$ are the internal torques along the tangential direction of the longitude and parallel circle under the action of the edge force *Q*_0_ and edge moment *M*_0_.

The internal force and internal torque equations of the elliptical head can be expressed as

8 $$ \left\{ {\begin{array}{*{20}l} {N_{\omega }^{{\left( {{\text{Q}}_{0} + {\text{M}}_{0} } \right)}} = N_{\omega }^{{{\text{Q}}_{0} }} = \tan \omega e^{{ - k_{1} \omega }} Q_{0} (\cos k_{1} \omega - \sin k_{1} \omega )R/R_{2} ,} \hfill \\ {N_{\uptheta }^{{({\text{Q}}_{0} + {\text{M}}_{0} )}} = N_{\uptheta }^{{{\text{Q}}_{0} }} = 0.25P{\text{Re}}^{{ - k_{1} \omega }} \cos k_{1} \omega (\text{a}/\text{b})^{2} \sqrt {R/R}_{2} ,} \hfill \\ {M_{\upomega }^{{\left( {{\text{Q}}_{0} + {\text{M}}_{0} } \right)}} = M_{\upomega }^{{{\text{Q}}_{0} }} = \frac{{e^{{ - k_{1} \omega }} Q_{0} R}}{{\sqrt[4]{{3(1 - \mu^{2} )}}}}\sqrt {\frac{t}{{R_{2} }}} \sin k_{1} \omega ,} \hfill \\ \begin{gathered}  M_{\uptheta }^{{({\text{Q}}_{0} + {\text{M}}_{0} )}} = M_{\uptheta }^{{{\text{Q}}_{0} }} = \mu M_{\omega }^{{{\text{Q}}_{0} }} , \hfill \\  k_{1} = \sqrt[4]{{3\left( {1 - \mu^{2} } \right)}}R_{1} /\sqrt {R_{2} t} , \hfill \\ R_{1} = (a^{4} y^{2} + b^{4} x^{2} )^{1.5} /(a^{4} b^{4} ), \hfill \\ R_{2} = (a^{4} y^{2} + b^{4} x^{2} )^{0.5} /b^{2} . \hfill \\ \end{gathered} \hfill \\ \end{array} } \right. $$

where *R*_1_ is the radius of the first curvature, *R*_2_ is the radius of the second curvature, *x* and *y* are the coordinates of the measurement point on the elliptical head, *a* is the long half shaft, and *b* is the short plate shaft.

Substituting Eq. (8) into Eq. (7) yields the total theoretical stress in the edge region of the elliptical head as follows:

9 $$ \left\{ {\begin{array}{*{20}l} {\sigma_{{{\upomega}}} = \frac{{PR_{2} }}{2t} + \frac{{P\tan \omega e^{{ - k_{1} \omega }} \sqrt {Rt} (\cos k_{1} \omega - \sin k_{1} \omega )\left( \frac{a}{b} \right)^{2} R}}{{8t\sqrt[4]{{3(1 - \mu^{2} )}}R_{2} }} \pm \frac{{3P{\text{Re}}^{{ - k_{1} \omega }} \sin k_{1} \omega }}{{4t\sqrt {3(1 - \mu^{2} )} }}\left( \frac{a}{b} \right)^{2} \sqrt {\frac{R}{{R_{2} }}} ,} \hfill \\ {\sigma_{{{\uptheta}}} = \frac{{PR_{2} }}{2t}\left( {2 - \frac{{R_{2} }}{{R_{1} }}} \right) + \frac{{P{\text{Re}}^{{ - k_{1} \omega }} \cos k_{1} \omega }}{4t}\left( \frac{a}{b} \right)^{2} \sqrt {\frac{R}{{R_{2} }}} \pm \frac{{6PRt\mu e^{{ - k_{1} \omega }} \sin k_{1} \omega }}{{8t^{2} \sqrt {3(1 - \mu^{2} )} }}\left( \frac{a}{b} \right)^{2} \sqrt {\frac{R}{{R_{2} }}} .} \hfill \\ \end{array} } \right. $$

The theoretical stress equation of the cylinder is expressed as

10 $$ \left\{ {\begin{array}{*{20}c} {\sigma_{\text{x}} = \sigma_{\text{x}}^{{\text{P}}} + N_{\text{x}}^{{({\text{Q}}_{0} + {\text{M}}_{0} )}} /t \pm 6M_{\text{x}}^{{({\text{Q}}_{0} + {\text{M}}_{0} )}} /t^{2} ,} \\ {\sigma_{\uptheta } = \sigma_{\uptheta }^{{\text{P}}} + N_{\uptheta }^{{({\text{Q}}_{0} + {\text{M}}_{0} )}} /t \pm 6M_{\uptheta }^{{({\text{Q}}_{0} + {\text{M}}_{0} )}} /t^{2} .} \\ \end{array} } \right. $$

where subscripts *θ* and *x* correspond to the tangential direction of the parallel circle and longitude, respectively. *σ*_x_ and *σ*_θ_ represent the total longitudinal stress and circumferential stress, respectively. $\sigma_{{\varvec{x}}}^{{\varvec{P}}}$ and $\sigma_{\uptheta }^{{\varvec{P}}}$ represent the longitudinal stress and circumferential stress under internal pressure *P*, respectively. $N_{{\text{x}}}^{{{\text{Q}}_{0} }}$, $N_{\uptheta }^{{{\text{Q}}_{0} }}$, $N_{{\text{x}}}^{{{\text{M}}_{0} }}$, and $N_{\uptheta }^{{{\text{M}}_{0} }}$ denote the internal forces along the tangential direction of the longitude and parallel circle under the action of the edge force *Q*_0_ and edge moment *M*_0_. $M_{{\text{x}}}^{{{\text{Q}}_{0} }}$, $M_{\uptheta }^{{{\text{Q}}_{0} }}$, $M_{{\text{x}}}^{{{\text{M}}_{0} }}$, and $M_{\uptheta }^{{{\text{M}}_{0} }}$ denote the internal torque along the tangential direction of the longitude and parallel circle under the action of the edge force *Q*_0_ and edge moment *M*_0_.

The internal force and internal torque equations of the cylinder can be expressed as

11 $$ \left\{ {\begin{array}{*{20}l} {N_{{\text{x}}}^{{\left( {{\text{Q}}_{0} + {\text{M}}_{0} } \right)}} = N_{{\text{x}}}^{{{\text{Q}}_{0} }} = 0,} \hfill \\ {N_{\uptheta }^{{({\text{Q}}_{0} + {\text{M}}_{0} )}} = N_{\uptheta }^{{{\text{Q}}_{0} }} = - 2k{\text{Re}}^{ - kx} Q_{0} \cos kx,} \hfill \\ {M_{{\text{x}}}^{{({\text{Q}}_{0} + {\text{M}}_{0} )}} = - e^{ - kx} Q_{0} \sin kx/k,} \hfill \\ \begin{gathered} M_{\uptheta }^{{({\text{Q}}_{0} + {\text{M}}_{0} )}} = \mu M_{x}^{{(\text{Q}_{0} + \text{M}_{0} )}} , \hfill \\ k = \sqrt[4]{{3\left( {1 - \mu^{2} } \right)}}/\sqrt {Rt} . \hfill \\ \end{gathered} \hfill \\ \end{array} } \right. $$

Substituting Eq. (11) into Eq. (10) yields the total theoretical stress in the edge region of the cylinder.

## Stress analysis and verification in the discontinuous region between the elliptical head and cylinder

To verify the correctness of the theoretical stress in the discontinuous region between the elliptical head and cylinder, the experimental stress of the elliptical head under different pressures is investigated using an internal-pressure thin-walled vessel stress measurement experimental device. The accuracy of the theoretical and experimental stress values in the discontinuous region is verified by comparing the errors of the theoretical and experimental stress values to provide reference for the stress distribution of the elliptical head.

### Measurement of experimental stress in the discontinuous region of the elliptical head

The material and mechanical properties of the prototype for the experiment are listed in Table 1.

**Table 1:** Material and mechanical properties of the prototype.

Property	Value
inner radius *R*/mm	200
thickness *t*/mm	4
material	0Cr18Ni9
Poisson’s ratio *μ*	0.3
elastic modulus *E*/GPa	206

**Figure 4:**
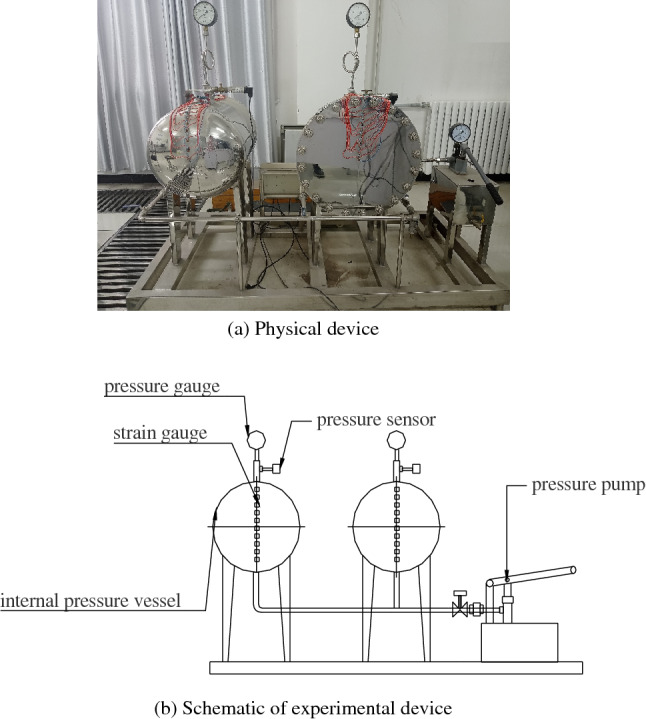
Internal pressure thin-walled vessel stress measurement experimental device.

**Figure 5:**
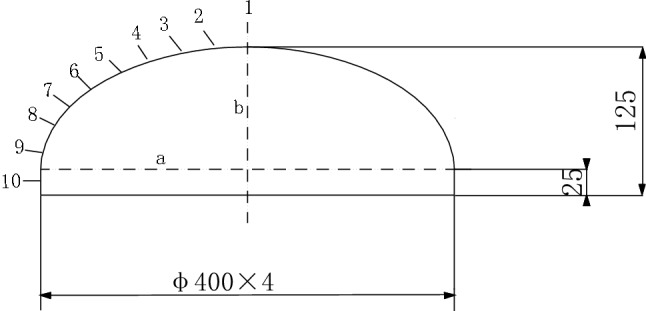
Distribution of measurement points.

The experimental device is shown in Fig. 4. First, water is added to the pressure pump, and by shaking the pressure pump longitudinally, the pipeline is pressurized. The pressure is transmitted to the internal pressure vessel to produce stress and strain. The stress is measured using a pressure gauge and transmitted to a computer through a pressure sensor. The strain is measured using a strain gauge. Ten strain gauge measurement points are set. The distance from each measurement point to the vertex of the elliptical head is shown in Table 2. Measurement points 1 to 9 are located on the elliptical head, measurement point 10 is located on the cylinder, and the distribution of the measurement points is shown in Fig. 5.

**Table 2:** Distance from measurement point to the vertex of the elliptical head (mm).

Measurement point	1	2	3	4	5	6	7	8	9	10
Distance	0	40	80	120	150	170	190	210	230	250

**Figure 6:**
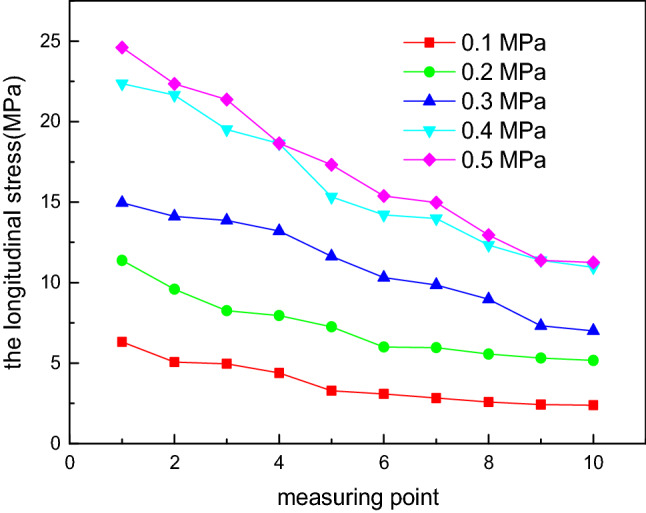
Influence of the internal pressure on the experimental values of the longitudinal stress at each measurement point.

**Figure 7:**
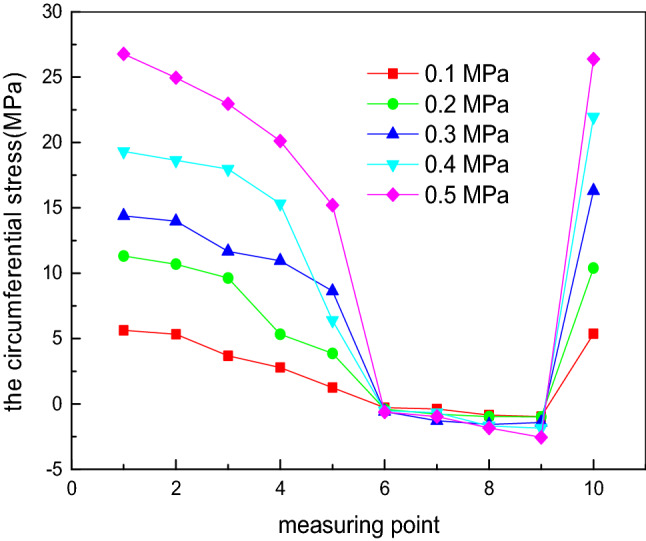
Influence of the internal pressure on the experimental values of the circumferential stress at each measurement point.

Considering constant geometric dimensions of the elliptical head, the internal pressure *P* value is set as 0.1 MPa, 0.2 MPa, 0.3 MPa, 0.4 MPa and 0.5 MPa. By changing the internal pressure *P*, the influence of the internal pressure on the experimental values of the longitudinal stress and circumferential stress at each measurement point is investigated, as shown in Figs. 6 and 7. When the internal pressure *P* is unchanged, the longitudinal stress value is always greater than zero, corresponding to tensile stress. Measurement point 1 is located at the vertex, and measurement point 10 is located at the equator. The longitudinal stress gradually decreases from measurement points 1 to 10. The longitudinal stress is the maximum at measurement point 1, and the longitudinal stress at measurement point 10 is half the stress value at measurement point 1. The reduction range from measurement points 8 to 10 is less because these measurement points are located in the discontinuous region, which bears not only the membrane stress but also the discontinuous stress. In terms of the circumferential stress, the variation trend is more complex and can be divided into three stages^33^. In the first stage, from measurement points 1 to 6, the circumferential stress is greater than zero, corresponding to tensile stress; in the second stage, from measurement points 7 to 9, the circumferential stress is less than zero, corresponding to compressive stress; in the third stage, the circumferential stress is greater than zero at measurement point 10, corresponding to tensile stress. From measurement points 7 to 10, the stress first decreases and later increases, similar to the longitudinal stress, affected by the discontinuous stress^34^. At measurement point 1, the longitudinal stress and circumferential stress are approximately equal. At measurement point 6, the circumferential stress changes from tensile stress to compressive stress. This phenomenon occurs because the stress distribution of the elliptical head is complex. The value of the longitudinal stress is always greater than zero, corresponding to tensile stress, and it gradually decreases from the vertex to the equator. The circumferential stress is related to a/b. When a/b < 2^0.5^, the stress value is always greater than zero, corresponding to tensile stress, and this value gradually decreases from the vertex to the equator. When a/b = 2^0.5^, the value is zero at the equator, and when a/b > 2^0.5^, compressive stress occurs. In this study, the a/b value of the experimental device is 2, and thus, the longitudinal stress is always tensile stress, and the circumferential stress is compressive stress^35–38^.

### Calculation of the theoretical stress in the discontinuous region of the elliptical head

According to the arc length formula, the meridian angles (radian system) of measurement points 1 to 9 starting from the boundary are 1.57, 1.43, 0.62, 0.60, 0.43, 0.33, 0.23, 0.11 and 0.06, respectively. Measurement point 10 is distributed on the cylinder. According to the conversion, the distance in the meridian direction from the boundary of measurement point 10 is 10 mm. The distances between the measurement points and edge of the elliptical head are listed in Table 3.

**Table 3:** Distance between measurement point and edge of the elliptical head (mm).

Measurement point	1	2	3	4	5	6	7	8	9
*R*_1_	400	382	260	253	185	138	96	61	53
*R*_2_	400	394	347	343	309	281	248	214	204

The stress enhancement coefficient of each measurement point, calculated using the abovementioned formula, is presented in Table 4:

**Table 4:** Stress enhancement coefficient for each measurement point.

Measurement point	1	2	3	4	5	6	7	8	9
*K*	5142	4875	3117	3015	2086	1491	968	577	491

**Figure 8:**
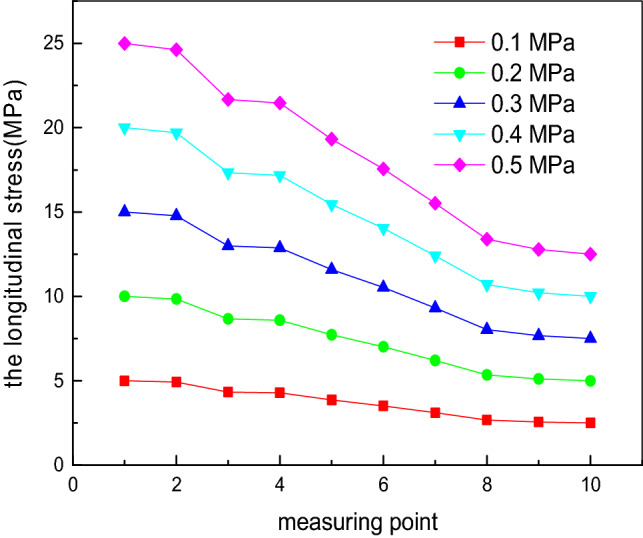
Influence of the internal pressure on the theoretical values of the longitudinal stress at each measurement point.

**Figure 9:**
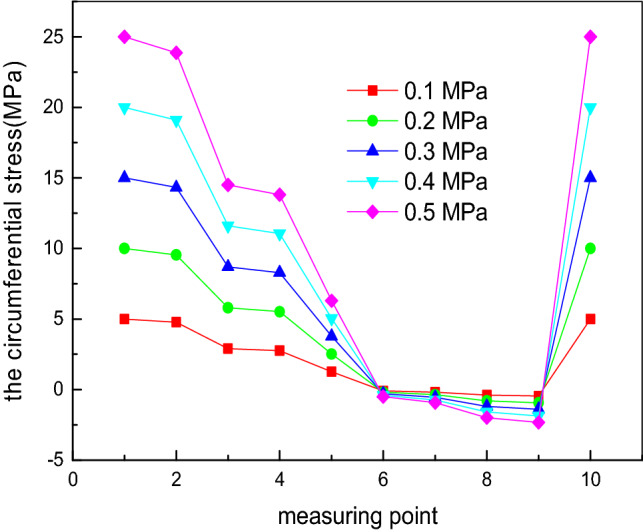
Influence of the internal pressure on the theoretical values of the circumferential stress at each measurement point.

By substituting these data into the total stress calculation formula of the elliptical head and cylinder edge region, the theoretical longitudinal stress and circumferential stress at measurement points 1 to 10 can be obtained, as shown in Figs. 8 and 9. The findings are consistent with the variation law of the experimental stress: For the same internal pressure *P*, the theoretical longitudinal stress is always greater than zero, corresponding to tensile stress, and its value gradually decreases from measurement points 1 (vertex) to 10 (equator). The theoretical circumferential stress is compressive stress^39^. At the vertex, the theoretical longitudinal stress is approximately equal to the theoretical circumferential stress^40^.

### Comparison of two analysis methods for the elliptical head

**Figure 10:**
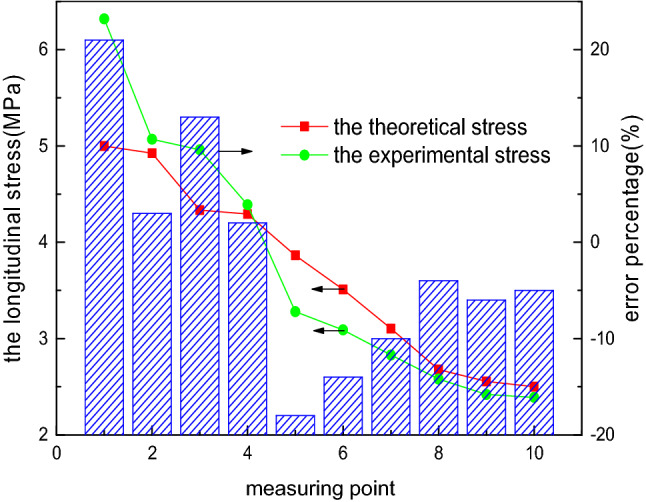
Longitudinal stress obtained using the two analysis methods.

**Figure 11:**
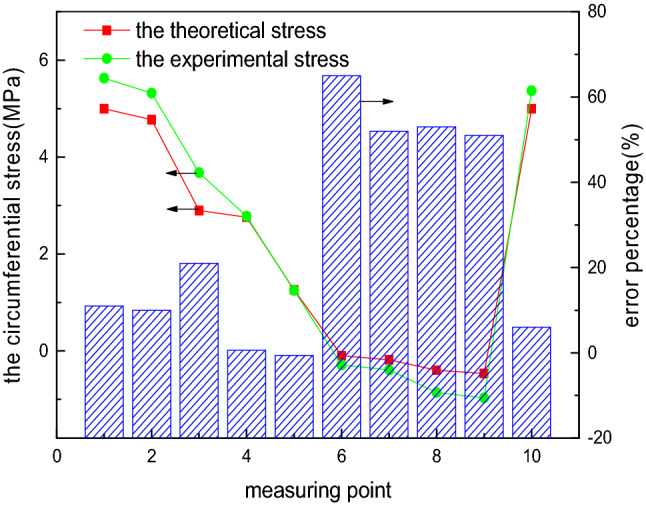
Circumferential stress obtained using the two analysis methods.

To compare the accuracy of the two analysis methods of the elliptical head, the internal pressure load of 0.1 MPa is considered as an example, and the comparison of the theoretical stress and experimental stress is presented in Figs. 10 and 11. The variation law of the theoretical stress of the elliptical head is consistent with that of the experimental stress, which verifies the correctness and reliability of the theoretical method. For the longitudinal stress, a large error is noted between the theoretical and experimental values at measurement point 1, which can be attributed to the inaccurate location of the strain gauge or sensitivity of the temperature and test instrument.

### Finite element analysis of the elliptical head

**Figure 12:**
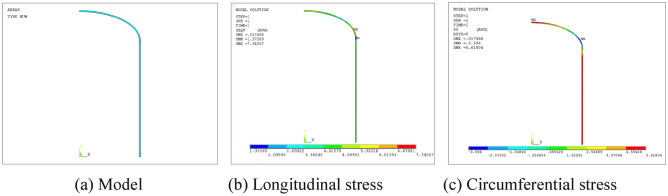
Finite element analysis of the elliptical head.

Considering the elliptical head as the research object, an FEM analysis is conducted using ANSYS software under an internal pressure load of 0.1 The model is shown in Fig. 12a, and the distributions of the longitudinal stress and circumferential stress are shown in Fig. 12b,c, respectively. The longitudinal stress is always greater than zero, corresponding to tensile stress. The circumferential stress decreases gradually from the apex to the equator, and compressive stress occurs.

**Figure 13:**
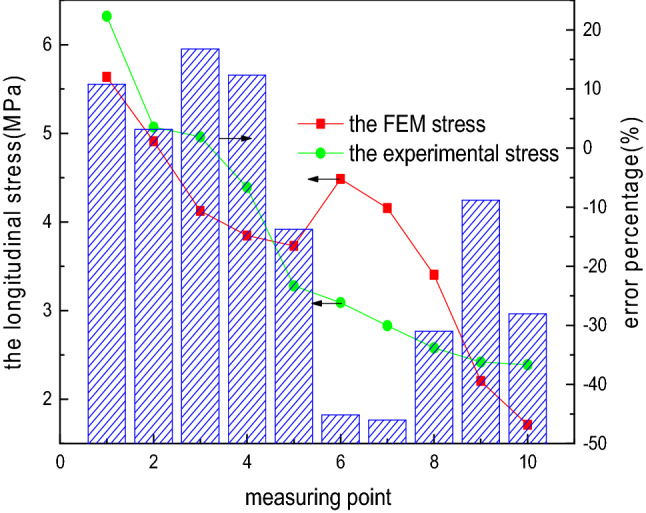
FEM-obtained longitudinal stress of the elliptical head.

**Figure 14:**
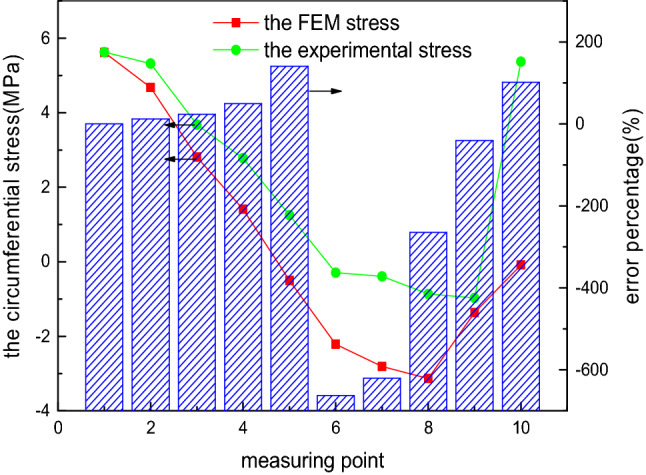
FEM-obtained circumferential stress of the elliptical head.

To compare the accuracy of the FEM-obtained stress and experimental stress of the elliptical head, the internal pressure load of 0.1 MPa is considered as an example, and the comparison of the FEM-obtained stress and experimental stress is shown in Figs. 13 and 14. The FEM-obtained longitudinal stress first decreases, subsequently increases, and later gradually decreases from measurement points 1 to 10. This phenomenon is highly inconsistent with the variation trend of the experimental longitudinal stress, and the relative error percentage is up to 45%. The FEM circumferential stress first decreases and later increases from measurement points 1 to 10, and compressive stress occurs, consistent with the change trend of the experimental longitudinal stress. However, the relative error percentage is extremely large, up to even 600%. The comparison results show that the theoretical formula is superior to the FEM for solving this problem.

## Conclusion

1. The theoretical stress calculation formula for the discontinuous region between the elliptical head and cylinder is obtained considering the deformation continuity equation, edge force, edge moment, internal force and internal moment.
2. When the internal pressure load is constant, the theoretical meridian stress is always greater than zero, corresponding to tensile stress, and this value decreases gradually from the vertex to the equator. The variation trend for the theoretical circumferential is relatively complex and can be divided into three stages; moreover, this stress is compressive. At the vertex, the longitudinal stress and circumferential stress are approximately equal. The change from measurement points 8 to 10 is influenced by the discontinuous stress, and the variation trend is abrupt.
3. By comparing and analysing the theoretical stress and experimental stress in the discontinuous region between the elliptical head and cylinder, the accuracy and applicability of the theoretical stress calculation formula in the discontinuous region are verified.
